# Comparison of mortality risk evaluation tools efficacy in critically ill COVID-19 patients

**DOI:** 10.1186/s12879-021-06866-2

**Published:** 2021-11-22

**Authors:** Vaidas Vicka, Elija Januskeviciute, Sigute Miskinyte, Donata Ringaitiene, Mindaugas Serpytis, Andrius Klimasauskas, Ligita Jancoriene, Jurate Sipylaite

**Affiliations:** 1 Clinic of Anaesthesiology and Intensive Care, Institute of Clinical Medicine, Faculty of Medicine, Vilnius University, Vilnius, Lithuania; 2 Faculty of Medicine, Vilnius University, Vilnius, Lithuania; 3 Institute of Clinical Medicine, Faculty of Medicine, Vilnius University, Vilnius, Lithuania

**Keywords:** ICU, Risk scores, Mortality, COVID-19

## Abstract

**Background:**

As the COVID-19 pandemic continues, the number of patients admitted to the intensive care unit (ICU) is still increasing. The aim of our article is to estimate which of the conventional ICU mortality risk scores is the most accurate at predicting mortality in COVID-19 patients and to determine how these scores can be used in combination with the 4C Mortality Score.

**Methods:**

This was a retrospective study of critically ill COVID-19 patients treated in tertiary reference COVID-19 hospitals during the year 2020. The 4C Mortality Score was calculated upon admission to the hospital. The Simplified Acute Physiology Score (SAPS) II, Acute Physiology and Chronic Health Evaluation (APACHE) II, and Sequential Organ Failure Assessment (SOFA) scores were calculated upon admission to the ICU. Patients were divided into two groups: ICU survivors and ICU non-survivors.

**Results:**

A total of 249 patients were included in the study, of which 63.1% were male. The average age of all patients was 61.32 ± 13.3 years. The all-cause ICU mortality ratio was 41.4% (n = 103). To determine the accuracy of the ICU mortality risk scores a ROC-AUC analysis was performed. The most accurate scale was the APACHE II, with an AUC value of 0.772 (95% CI 0.714–0.830; p < 0.001). All of the ICU risk scores and 4C Mortality Score were significant mortality predictors in the univariate regression analysis. The multivariate regression analysis was completed to elucidate which of the scores can be used in combination with the independent predictive value. In the final model, the APACHE II and 4C Mortality Score prevailed. For each point increase in the APACHE II, mortality risk increased by 1.155 (OR 1.155, 95% CI 1.085–1.229; p < 0.001), and for each point increase in the 4C Mortality Score, mortality risk increased by 1.191 (OR 1.191, 95% CI 1.086–1.306; p < 0.001), demonstrating the best overall calibration of the model.

**Conclusions:**

The study demonstrated that the APACHE II had the best discrimination of mortality in ICU patients. Both the APACHE II and 4C Mortality Score independently predict mortality risk and can be used concomitantly.

## Background

As the SARS-CoV-2 (COVID-19) pandemic has continued in the year 2020, the number of COVID-19 patients admitted to the intensive care unit (ICU) around the world has severely increased. According to several reports, over 20% of patients hospitalized with COVID-19 are admitted to the ICU [1]. Despite working at their maximum capacity and increasing the number of beds and personnel, these services are overstretched, leading to worse clinical outcomes [2]. Having regard for the collapsing health care systems, this might raise the question of triage criteria amendments, since not all patients can or should be admitted to the ICU [3–5]. Furthermore, the strategy of admission to the ICU varies in different countries. For example, in Belgium, more patients died in the wards (72%) than in the ICU (28%) [6]. The chances of survival are affected by many variables, including the diagnosis upon admission, the patient’s comorbidities, the severity of organ failure, the patient’s age and the patient’s health status before admission. Therefore, it is critical to triage the patients vigorously, determining which patients have the best chances of successful treatment [7].

There are several scores used in the ICU to help clinicians estimate the mortality risk of patients. Three of the most common are the Simplified Acute Physiology Score (SAPS) II, Acute Physiology and Chronic Health Evaluation (APACHE) II and Sequential Organ Failure Assessment (SOFA) score. The SOFA score uses clinical parameters and laboratory values, while the SAPS II and APACHE II also include age, history of severe organ failure or chronic disease and type of admission. The APACHE II and SAPS II should be calculated on newly admitted patients, while the SOFA score can be recalculated every 24 h [8]. All of these scales have perfect calibration and discrimination across all ranges of possible values, determining the risk of mortality from 1% to almost up to 100%. However, it is important to note that the APACHE II was originally developed to fit all kinds of ICU populations. Therefore, it might not be as precise when evaluating specific patient groups and individual patients. The SAPS II has been built in an European/North American environment, which is important while evaluating patients on different continents [9, 10]. Furthermore, all these scores are focused on a momentary evaluation and do not give enough attention to the previous state of the patient, both chronic comorbidities and ongoing decompensation. As we have established, none of these scores are perfect in every setting, and none of them is specific to one illness, being less accurate in patients which have a particular disease.

Since the beginning of the COVID-19 pandemic, the patients with the highest mortality were those with chronic diseases, such as arterial hypertension, diabetes, heart failure, obesity and chronic kidney disease [11]. Due to these particularities, COVID-19 merits a risk stratification model of its own. Thus, the 4C Mortality Score was developed in the year 2020 in the middle of the pandemic. Using eight different parameters to evaluate patients, it was originally tested in a population that was fully Caucasian, 43% male, had a mean age of 73 and mortality rate of 32.3% [12]. The 4C Mortality Score is designed to be implemented at the moment of the hospitalization and was not intended to be used when admitting the patient to the ICU. However, it is highly specific to COVID-19, as it encompasses the parameters that are the most critical in this disease (i.e., those that reflect respiratory function and inflammatory processes) and patient demographics and comorbidities. It is likely that the effect of the 4C Mortality Score determinants remain present during treatment of the patients, aiding in deterioration, transfer to the ICU and, sequentially, mortality of the population.

The aim of this study was to estimate which of the conventional ICU mortality risk scores is the most accurate at predicting mortality in COVID-19 patients and to determine how can these scores be used in combination with the 4C Mortality Score.

## Methods

### Study population

This was a retrospective study of patients who were admitted to a tertiary referral university hospital in the year of 2020 and tested positive for SARS-CoV-2. Ethical approval was gained from the Regional Research Ethics Committee to conduct the study. Inclusion criteria were as follows: tested positive for SARS-CoV-2 infection, 18 years or older and admitted to the intensive care unit (ICU).

### Mortality risk evaluation

The patients were evaluated two times during the study. Upon admission to the hospital, the 4C Mortality Score was calculated. Upon admission to the ICU, the APACHE II, SAPS II and SOFA scores were implemented. All the scores were used as suggested by the creators of the scores [9, 10, 12, 13].

### Definition of the outcome and groups in the study

Mortality was set as all-cause mortality in the ICU. All the cases in the study had been either discharged or deceased during collection of the data. Mortality ratios were calculated and standardized according to the recommendations of the authors of the risk evaluation tools. The patients were split into two groups: ICU survivors and ICU non-survivors.

### Statistical analysis

#### Descriptive analysis

Statistical analysis was carried out by the SPSS statistical software package version 26.0 (IBM/SPSS, Inc., Chicago, IL). Baseline characteristics were defined using descriptive statistics. Categorical variables were stated as an absolute number (n) and a relative frequency (%), and continuous variables were represented as a median (interquartile range) or as a mean (± SD), depending on the normality of the distribution. The normality of distribution was tested by the one sample Kolmogorov–Smirnov test.

#### Comparison of survivors and non-survivors

To compare the categorical variables, the chi-square test was performed. To compare the continuous variables, the independent samples t-test was used for the normally distributed data and the Mann–Whitney test for the non-parametric data.

#### Standardized mortality ratio calculation

The standardized mortality ratio (SMR) represents the excess mortality and was calculated using the observed number of lethal cases divided by the predicted number of lethal cases. The observed count was obtained from the study data. The predicted number was obtained by implementing the percentage of mortality risk from all the tools used (APACHE II, SOFA, SAPS II and 4C Mortality Score). The individual values of the risk scores were averaged to represent the study population.

#### Accuracy testing

The receiver operating characteristic area under the curves (ROC-AUCs) were examined to identify the accuracy of discrimination of the APACHE II, SAPS II, SOFA and 4C Mortality Score.

#### Regression analysis

To determine the independent predictive value of all the risk scores, these scores were integrated into a forward logistic regression analysis. The Lemeshow–Hosmer goodness-of-fit test was used to evaluate calibration.

## Results

### Baseline characteristics

A total of 249 patients were included in the study, of which 63.1% were male. The mean age of the patients was 61.32 ± 13.3 years. Most of the patients were aged 50–70 years old (55.4%). The highest mortality was observed in the > 80 years age group (63.6%). The most common comorbidities were obesity (28.9%), hypertension (75.9%), and chronic cardiac disease (46.6%) (Table 1).

**Table 1:** Baseline characteristics of the patients

Variable	All sample, n = 249	Survivors, n = 146 (58.6%)	Non-survivors, n = 103 (41.4%)	p value
*Demographics*				
Gender				
Female	92 (36.9)	52 (35.6)	40 (38.8)	0.604
Male	157 (63.1)	94 (64.4)	63 (61.2)	
Age (years)	61.32 ± 13.30	57.74 ± 13.60	66.39 ± 11.09	< 0.001
Age groups (% within group)				
< 50 years	42 (16.9)	35 (83.3)	7 (16.7)	
50–59 years	75 (30.1)	48 (64.0)	27 (36.0)	
60–69 years	63 (25.3)	36 (57.1)	27 (42.9)	
70–79 years	47 (18.9)	19 (40.4)	28 (59.6)	
> 80 years	22 (8.8)	8 (36.4)	14 (63.6)	
*Comorbidities*				
Obesity	72 (28.9)	43 (29.5)	29 (28.2)	0.824
Hypertension	189 (75.9)	103 (70.5)	86 (83.5)	0.019
Chronic cardiac disease	116 (46.6)	55 (37.7)	61 (59.2)	0.001
CKD	87 (34.9)	38 (26.0)	49 (47.6)	< 0.001
Immunosuppression	30 (12.0)	17 (11.6)	13 (12.6)	0.815
Diabetes	81 (32.5)	51 (34.9)	30 (29.1)	0.336
COPD	26 (10.4)	13 (8.9)	13 (12.6)	0.345
Asthma	13 (5.2)	8 (5.5)	5 (4.9)	0.827
*Clinical signs upon admission*				
Fever	36.8 [36.6–37.4]	36.9 [36.6–37.4]	36.7 [36.5–37.5]	0.169
MAP	93.1 ± 16.2	94.5 ± 13.0	91.2 ± 19.9	0.117
Heart rate	84 [72–100]	81 [72–94]	88.5 [74–104.75]	0.043
RR	22 [20–27]	22 [18–26]	25 [20–30]	0.001
SpO_2_	91 [86–95]	93 [89–96]	88 [82–93]	< 0.001
PaO_2_/FiO_2_	123.6 [70.7–192.0]	161.5 [80.2–217.8]	84.0 [59.8–146.0]	< 0.001
*Mortality risk scores*				
SOFA	3 [2–6]	3 [2–5]	5 [3–9]	< 0.001
Mortality risk % (SOFA)	10 [3.8–4.5]	10 [3.8–10]	10 [3.3–22.5]	
SMR (SOFA)	4.14			
SAPS II	25 [18–34]	21 [16–29]	32 [24–41]	< 0.001
Mortality risk % (SAPS II)	10 [10–10]	10 [10–10]	25 [10–25]	
SMR (SAPS II)	4.14			
APACHE II	12 [9–16]	10 [7–13]	15 [11–15]	< 0.001
Mortality risk % (APACHE II)	14.6 [9.9–23.5]	11.3 [7.6–16.5]	21 [12.9–21]	
SMR (APACHE II)	2.84			
4C Mortality	10 [7–13]	8 [6–11]	12 [9–15]	< 0.001
Mortality risk % (4C Mortality)	39.3 [7.7–52.1]	7.7 [4.5–44.5]	44.5[39.3–61.5]	
SMR (4C Mortality)	1.05			
*Clinical course*				
MV	117 (47.0)	80 (77.7)	37 (25.3)	< 0.001
AKI	111 (44.6)	83 (80.6)	28 (19.2)	< 0.001
LOS in ICU	9 [4–16]	7 [4–14]	13 [5–17]	0.01
LOS in hospital	17 [12–28]	22 [14–31]	15 [9.75–20.25]	< 0.001

*CKD* chronic kidney disease, *COPD* chronic obstructive pulmonary disease, *MAP* mean arterial pressure, *RR* respiratory rate, *MV* mechanical ventilation, *AKI* acute kidney injury, *ICU* intensive care unit, *SAPS II* Simplified Acute Physiology Score II, *SOFA* Sequential Organ Failure Assessment, *APACHE II* Acute Physiology and Chronic Health Evaluation II, *4C Mortality* 4C Mortality Score, *SMR* standardized mortality ratio, *LOS* length of stay

SMRs were calculated, revealing several times higher values for the APACHE II (SMR = 2.84), SOFA (SMR = 4.14) and SAPS II (SMR = 4.14). The SMR of the 4C Mortality Score was 1.05, showing a good concordance to the actual mortality rate of the group (Table 1).

The mean values of the mortality risk scores were higher in the ICU non-survivors group: SOFA, 3 [2–5] vs 5 [3–9] (p < 0.001); SAPS II, 21 [16–29] vs 32 [24–41] (p < 0.001); APACHE II, 10 [7–13] vs 15 [11–15] (p < 0.001); and 4C Mortality Score, 8 [6–11] vs 12 [9–15] (p < 0.001) (Table 1).

Moreover, the PaO_2_/FiO_2_ ratio was also lower in the ICU non-survivors group: 84 [59.8–146.0] vs 161.5 [80.2–217.8] (p < 0.001). The average length of stay in the ICU of all patients was 9 days and the overall length of stay in the hospital was 17 days (Table 1).

### Accuracy of mortality risk scores

**Fig. 1:**
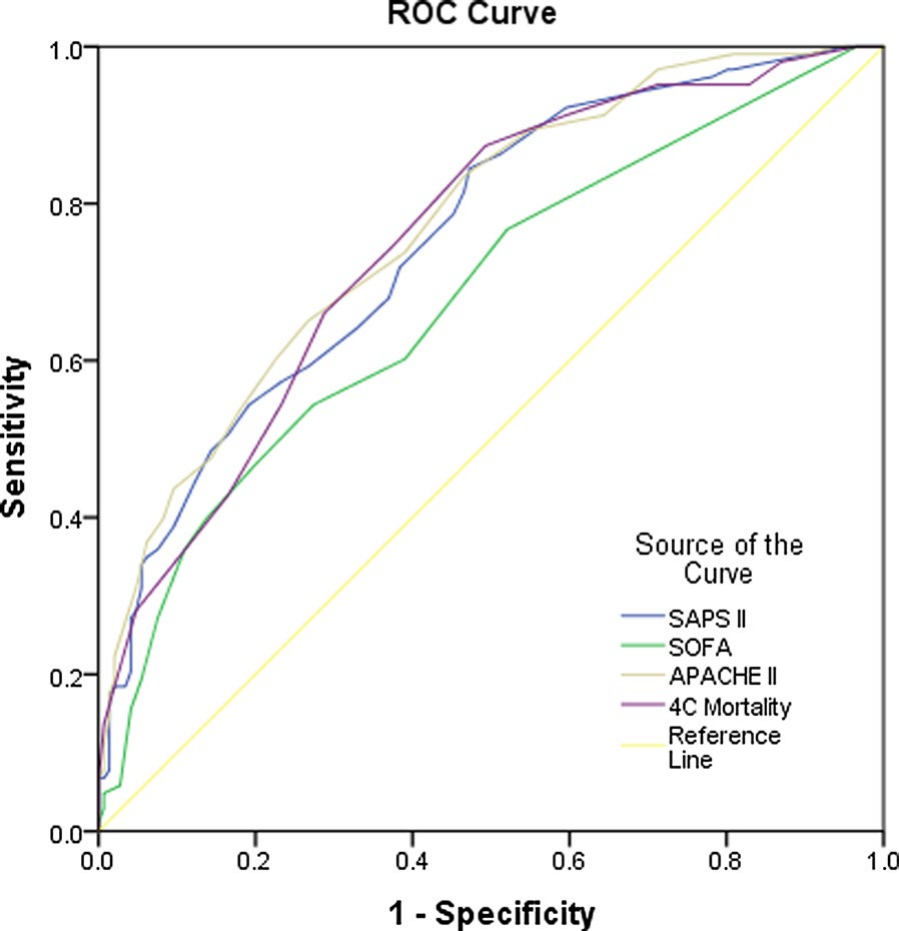
Accuracy of mortality risk scores. The ROC-AUCs for the mortality risk scores in the ICU and 4C Mortality Score reference lines denote the null hypothesis that the AUC really equals 0.50. *ICU* intensive care unit, *ROC-AUC* receiver operating characteristic area under the curve, *SAPS II* Simplified Acute Physiology Score II, *SOFA* Sequential Organ Failure Assessment, *APACHE II* Acute Physiology and Chronic Health II, *4C Mortality* 4C Mortality Score

To determine their accuracy of discrimination, a ROC-AUC analysis of the ICU mortality risk scores was performed. All the risk scores were good predictors of mortality, generating ROC-AUC values above 0.5. The most accurate scale was the APACHE II, with an AUC value of 0.772 (95% CI 0.714–0.830; p < 0.001). The 4C Mortality Score had an AUC value of 0.754 (95% CI 0.694–0.814; p < 0.001). These results are presented in Fig. 1 and Table 2.

**Table 2:** Accuracy of mortality risk scores

Risk score	AUC	p value	95% CI	
SOFA	0.679	< 0.001	0.611	0.747
SAPS II	0.755	< 0.001	0.695	0.815
APACHE II	0.772	< 0.001	0.714	0.830
4C Mortality	0.754	< 0.001	0.694	0.814

The AUC values for each ROC curve for the ICU mortality risk scores and 4C Mortality Score, p-value denotes the null hypothesis that the AUC really equals 0.50

*ICU* intensive care unit, *ROC-AUC* receiver operating characteristic area under the curve, *SAPS II* Simplified Acute Physiology Score II, *SOFA* Sequential Organ Failure Assessment, *APACHE II* Acute Physiology and Chronic Health Evaluation II, *4C Mortality* 4C Mortality Score

### Regression analysis of mortality risk scores

Univariate regression analysis was performed to determine the link between the risk scores and ICU mortality. All of the ICU risk scores and 4C Mortality Score were significant mortality predictors in the analysis, with acceptable calibration. The results are presented in Table 3.

**Table 3:** Regression analysis of ICU mortality risk scores

Variable	Exp(B)	95% CI	p value
	*Univariate regression*		
APACHE II	1.210	1.142–1.283	< 0.001
4C Mortality	1.311	1.205–1.427	< 0.001
SAPS II	1.089	1.059–1.119	< 0.001
SOFA	1.216	1.120–1.321	< 0.001
	*Multivariate regression*		
APACHE II	1.155	1.085–1.229	< 0.001
4C Mortality	1.191	1.086–1.306	< 0.001
SAPS II	n.s	n.s	0.056
SOFA	n.s	n.s	0.141

Univariate and multivariate regression analysis of risk scores. All of the scores are significant predictors of mortality in the univariate regression, and the APACHE II and 4C Mortality Score persist in the multivariate model

*ICU* intensive care unit, *ROC-AUC* receiver operating characteristic area under the curve, *SAPS II* Simplified Acute Physiology Score II, *SOFA* Sequential Organ Failure Assessment, *APACHE II* Acute Physiology and Chronic Health II, *4C Mortality* 4C Mortality score, *n.s.* not significant

Multivariate regression analysis was performed to elucidate which of the scores can be used together with the independent predictive value. In the final model, the APACHE II and 4C Mortality Score prevailed, with the best fit of the model (χ^2^ = 4.72; degrees of freedom 8; p > 0.787). For each point increase in the APACHE II, mortality risk increased by 1.155 (OR 1.155, 95% CI 1.085–1.229; p < 0.001), and for each point increase in the 4C Mortality Score, mortality risk increased by 1.191 (OR 1.191, 95% CI 1.086–1.306; p < 0.001). Results are presented in Table 3. The R^2^ value of the final model was 0.358, suggesting a polymodal origin of the mortality in the ICU, which is supposed to not only be determined by the pre-hospitalization factors.

## Discussion

The main finding of the study is that the APACHE II score was the most accurate and had the best discrimination at predicting mortality risk in COVID-19 patients treated in the ICU. However, the best calibration was observed when the 4C Mortality Score was added to the model. Therefore, the APACHE II and 4C Mortality Score independently predict mortality risk and can be used concomitantly.

One of the main findings of the study is that conventional ICU mortality risk scores perform quite well in COVID-19 patients. In our study, the mean values of the APACHE II, SAPS II and SOFA scores are comparable with other reports. The overall mean APACHE II score in our study was 12, which is comparable to the 12.87 reported from India but lower than the ones reported from Sao Paolo (16.7) and Pakistan (20.84) [14–16]. Furthermore, in our study, the APACHE II score prevailed as the most accurate one with the highest AUC of all the scores (0.772) [17, 18]. These results correspond with the report from Sao Paolo (AUC 0.8). However, the overall accuracy is slightly lower than expected and reported in the literature [19]. Furthermore, the SMRs are several times higher than expected in these patients. This can be explained by the pathophysiology of the COVID-19 disease, which affects several organ systems far more extensively (i.e., respiratory and coagulation) in the beginning of the disease. Thus, the conventional scores, even when giving a maximum score in these dimensions, may under evaluate the overall mortality risk of these patients.

Secondly, the 4C Mortality Score fits into the risk prediction model with the APACHE II score for ICU patients. In studies comparing the risk scores developed specifically for COVID-19 patients, the 4C Mortality Score was the most accurate, with AUCs of 0.799 and 0.774, and 0.754 in our study. This score has a more extensive evaluation of the comorbidities of patients than conventional ICU risk scores, which tend to rely on stratifying the on-the-spot evaluation of the organ systems. In our study, the combination of the APACHE II and 4C Mortality Score increased the calibration of the risk determination model in the regression analysis [12, 18]. Despite this, the R^2^ value of 0.358 in the final model was only satisfactory when predicting the mortality in this group. It is obvious that the clinical course in the ICU and the application of mechanical ventilation, renal replacement therapy and other treatment options are major contributors to the outcome. Thus, further studies should be done either to elucidate more risk factors or to define the key moments in the treatment of this specific population.

It is important to discuss the potential clinical implementation of our findings. In the case of the pandemic and the overload of patients, the most valuable feature of the mortality score is good discriminative performance and pragmatic identification of the patients who are likely to die and will not benefit from treatment. Thus, the 4C Mortality Score is a perfect tool in the emergency department (ED). However, when evaluating the mortality of COVID-19 patients in the ICU, a more precise tool is needed. For example, since the 4C Mortality Score was developed for triage in the ED, a noninvasive oxygenation parameter (SpO_2_) was chosen to evaluate respiratory function. Pulse oximetric saturation has a good correlation with PaO_2_ in the range of 80–100%. However, critically ill COVID-19 patients usually stay in the ward until SpO_2_ levels drop below 80%. The accuracy of SpO_2_ drops when the SpO_2_ level is below 80% [20]. Moreover, in the case of the APACHE II score, it has a potentially better evaluation of chronic respiratory failure because of the inclusion of base excess. Furthermore, SpO_2_ strongly depends on blood flow, pulsatility and microcirculatory disturbances (such as microthrombosis), which can affect the accuracy of SpO_2_. Moreover, the APACHE II and SAPS II scores include a lot of parameters that are usually normal on admission in COVID-19 patients, but they may deteriorate during the clinical course of these patients, leading to high prognostic value when admitting patients to the ICU. Thus, we conclude that there is a benefit to combine the use of these scores. We suggest using the 4C Mortality Score when admitting patients to the hospital and to consider it when accepting the patient to the ICU, alongside the calculation of the APACHE II score.

This study is retrospective. Therefore, all the assumptions should be regarded as associations rather than as causations. Furthermore, it is important to consider the sample size of our study and keep in mind that, to get more generalizable results, more patients should be included in the study. There is a slight difference in the age of the patients in the study when compared with the literature. The mean age of the patients in our study was 61.32 years, which is an average number compared to several other studies with mean ages of 50, 51.26 and 74 years [14, 16, 18]. However, the proportion of patients over 70 years of age is quite large, almost 30%. Increasing age is known to be associated with higher mortality risk and poor outcome. This and other differences between the population of our study and the studies that validated the risk scores (ethnic, demographic, cultural and economic conditions of the patients) should also be considered.

This was a study with a pragmatic approach, having its design oriented towards clinical decision-making. If a patient in the ED met the criteria for hospitalization, he/she was evaluated with the 4C Mortality Score, independently whether he/she was hospitalized on a general ward or directly to the ICU. If the patient was transferred to the ICU, either directly or later, he/she was evaluated with ICU risk scores (APACHE II, SOFA and SAPS II). Therefore, the study design has a potential for bias, since the patient from the general ward may deteriorate and be transferred in a more grave state as compared to the state he/she was hospitalized and evaluated with the 4C Mortality Score. However, the 4C Mortality Score was not developed to use in patients that are already hospitalized. Thus, to offer a clinician a pragmatic way of risk evaluation, we designed this study with evaluations at two points in time, indeed, in some cases, occurring at the same time, when the patient is being admitted directly to the ICU and, in some cases, at two different points in time.

## Conclusion

The main finding of the study is that the APACHE II score was the most accurate and had the best discrimination at predicting mortality risk in COVID-19 patients treated in the ICU. However, the best calibration was observed when the 4C Mortality Score was added to the model. Therefore, the APACHE II score and 4C Mortality Score independently predict mortality risk and can be used concomitantly.

## Data Availability

The dataset used during the current study is available from the corresponding author on reasonable request.

## Abbreviations

ICU: Intensive care unit
COVID-19: Coronavirus disease 2019
SAPS II: Simplified Acute Physiology Score II
APACHE II: Acute Physiology and Chronic Health Evaluation II
SOFA: Sequential Organ Failure Assessment
ROC-AUC: Receiver operating characteristic area under the curve
CKD: Chronic kidney disease
COPD: Chronic obstructive pulmonary disease
MAP: Mean arterial pressure
RR: Respiratory rate
MV: Mechanical ventilation
AKI: Acute kidney injury
